# Deciphering the genetic basis of male fertility in Italian Brown Swiss dairy cattle

**DOI:** 10.1038/s41598-022-14889-1

**Published:** 2022-06-22

**Authors:** Hendyel A. Pacheco, Attilio Rossoni, Alessio Cecchinato, Francisco Peñagaricano

**Affiliations:** 1 Department of Animal and Dairy Sciences, University of Wisconsin-Madison, Madison, WI 53706 USA; 2 Italian Brown Breeders Association, Bussolengo, 37012 Verona, Italy; 3 Department of Agronomy, Food, Natural Resources, Animals and Environment, University of Padova, 35020 Legnaro, Padua Italy

**Keywords:** Animal breeding, Genetic association study, Genetic markers, Heritable quantitative trait, Quantitative trait

## Abstract

Improving reproductive performance remains a major goal in dairy cattle worldwide. Service sire has been recognized as an important factor affecting herd fertility. The main objective of this study was to reveal the genetic basis of male fertility in Italian Brown Swiss dairy cattle. Dataset included 1102 Italian Brown Swiss bulls with sire conception rate records genotyped with 454k single nucleotide polymorphisms. The analysis included whole-genome scans and gene-set analyses to identify genomic regions, individual genes and genetic mechanisms affecting Brown Swiss bull fertility. One genomic region on BTA1 showed significant additive effects. This region harbors gene *RABL3* which is implicated cell proliferation and motility. Two genomic regions, located on BTA6 and BTA26, showed marked non-additive effects. These regions harbor genes, such as *WDR19* and *ADGRA1*, that are directly involved in male fertility, including sperm motility, acrosome reaction, and embryonic development. The gene-set analysis revealed functional terms related to cell adhesion, cellular signaling, cellular transport, immune system, and embryonic development. Remarkably, a gene-set analysis also including Holstein and Jersey data, revealed significant processes that are common to the three dairy breeds, including cell migration, cell–cell interaction, GTPase activity, and the immune function. Overall, this comprehensive study contributes to a better understanding of the genetic basis of male fertility in cattle. In addition, our findings may guide the development of novel genomic strategies for improving service sire fertility in Brown Swiss cattle.

## Introduction

Pregnancy establishment in cattle is a complex process that involves numerous consecutive events, including gametogenesis, fertilization, blastocyst formation, conceptus elongation, pregnancy recognition, and placenta development. All these consecutive events should be accomplished in a well-orchestrated manner to achieve a successful pregnancy. The relative importance of the maternal, paternal, and embryonic factors on pregnancy establishment is still largely unknown^1^. Most research has focused on female fertility, while male fertility has received less attention. It is worth noting that the service sire has a direct influence not only in the fertilization process but also in early embryo development. Indeed, Ortega and collaborators have recently proved that the reduced ability of low fertility bulls to establish pregnancy is multifactorial and encompasses sperm fertilizing ability, preimplantation embryonic development, and placenta and embryo development after conceptus elongation and pregnancy recognition^2^. Therefore, current evidence suggests that bull fertility should not be overlooked in breeding schemes aimed at improving cow reproductive performance.

There is increasing evidence that genetic factors explain part of the differences in conception rate among sires. Some semen production and quality traits, such as ejaculate volume, sperm concentration, sperm motility, and sperm viability, have moderate heritability^3,4^. In addition, several transcriptomic, proteomic, and metabolomic studies have discovered numerous differences between the spermatozoa of high-fertility and low-fertility bulls^5–7^. Furthermore, multiple genomic approaches have been used to identify genetic variants associated with bull fertility. Both candidate gene studies and whole-genome scans have identified genomic regions and positional candidate genes associated with male fertility in both beef and dairy cattle^8,9^. Overall, current evidence suggests that bull fertility is influenced by genetic factors, and hence it could be improved by genetic means. Additionally, the identification of biomarkers for bull fertility could assist farmers, breeders, and AI companies make accurate management and selection decisions, such as using or marketing semen from high-fertility bulls or early culling of predicted subfertile bull calves^10^.

The Brown Swiss breed, one of the oldest dairy breeds in the world, has a great significance for the dairy industry. The breed is raised worldwide due to its adaptability to different climates, including Europe, both North and South America, and the Middle East. Europe has the largest Brown Swiss population, which is led by Switzerland, Germany, and Italy. Interestingly, we recently developed a bull fertility evaluation in the Italian Brown Swiss population using cow field data^11^. We used almost 400k breeding records from 130k lactating cows and more than 1200 bulls. The evaluation model included factors related to the bull under evaluation, such as bull age, bull inbreeding, and AI organization, and factors associated with the cow that receives the dose of semen, including herd-year-season, cow’s age, parity, and milk yield. Notably, we revealed a substantial variation in conception rate among Brown Swiss bulls, with more than 20% conception rate difference between high-fertility and low-fertility bulls^11^.

The main objective of this study was to reveal the genetic basis of male fertility in the Italian Brown Swiss dairy cattle population. We performed alternative whole-genome scans and gene-set analyses to identify genomic regions, individual genes and genetic mechanisms affecting Brown Swiss bull fertility. We also compared the results obtained in Brown Swiss with those previously obtained in Holstein and Jersey. The identification of genes and gene networks affecting bull fertility could have multiple benefits, including better understanding of this complex trait in cattle and promote the development of novel tools to make enhanced management and selection decisions on male fertility in the Brown Swiss breed.

## Materials and methods

### Phenotypic and genotypic data

Phenotypic data consisted in sire conception rate (SCR) records from a total of 1102 Italian Brown Swiss bulls. These sire conception rate records were estimated using cow field data^11^. Briefly, we first evaluated cow pregnancy success (binary trait; 0 = failure, 1 = pregnancy) using factors related to both the bull under evaluation and the cow that receives the dose of semen. Factors related to the cow included lactation number, age, days in milk at breeding, and total milk yield as fixed effects, and herd-year-season and both cow additive genetic and permanent environmental as random effects. We then estimated sire conception rate using only the factors closely related to the bull. Factors related to the bull included age and inbreeding as fixed effects and AI company and service sire as random effects. Estimates of sire conception rate were calculated as deviation from the mean, which is set as 0, so each 1-point difference reflects 1% more conception rate. For bulls with multiple SCR records, the most reliable SCR record, i.e., the SCR record with most breedings, was used in this study. Sire conception rate values ranged from − 22.3 to 9.9%, with number of breeding ranged from 50 to 8110. Supplementary Table 1 reports the distribution of number of bulls per number of breedings. The use of at least 50 breedings in at least 5 different herd-year-season groups is a compromise between number of bulls in the dataset and the reliability of the bull fertility estimates. On the one hand, the reliability of the bull fertility estimates depends on the number of breedings used for the evaluation. On the other hand, increasing the minimum number of breedings limits the number of available bulls in the dataset, which harms subsequent genomic analyses. Thus, balancing size of the dataset and using reliable phenotypes is important.

Genotype data for 572,528 single nucleotide polymorphism (SNP) markers located in autosomal chromosomes were available for all the 1102 Italian Brown Swiss bulls with SCR records. This set of SNP markers is a subset of the markers available in the BovineHD Genotyping BeadChip (Illumina Inc.). The SNP information, including chromosome and position, was based on the bovine reference genome ARS-UCD-1.2. SNP markers that were monomorphic or with calling rate less than 95% were removed from the dataset. After quality control, a total of 454,556 SNP markers were retained for subsequent genomic analysis.

### Whole-genome scans: additive and non-additive effects

The relevance of additive and non-additive effects, namely dominance, recessive and overdominance effects, on service sire fertility was evaluated on a genome-wide scale using a two-step mixed-model-based approach^12,13^.

In the first step, the following model was fitted:

$$ {\mathbf{y}} = {\mathbf{Xb}} + {\mathbf{Zu}} + {\mathbf{e}} $$

where **y** is the vector of SCR records, **b** is the vector of fixed effects, **u** is the vector of random animal effects, and **e** is the vector of random residual effects. The incidence matrices **X** and **Z** relate phenotypic records to fixed and animal effects, respectively. The random effects were assumed to follow a multivariate normal distribution with ${\mathbf{u}}\sim N(0,\;{\mathbf{G}}\sigma_{u}^{2} )$ and ${\mathbf{e}}\sim N(0,\;{\mathbf{I}}\sigma_{e}^{2} )$, where $\sigma_{u}^{2}$ and $\sigma_{e}^{2}$ are the animal additive genetic and residual variances respectively; **G** is the genomic relationship matrix, and **I** an identity matrix. The variance–covariance matrix for this animal model was estimated as ${\mathbf{V}}_{0} = {\mathbf{ZGZ}}^{\prime}\sigma_{u}^{2} + {\mathbf{I}}\sigma_{e}^{2}$.

In the second step, the following model was fitted for every SNP:

$${\mathbf{y}} = {\mathbf{X}}{\varvec{\upbeta }} + X_{SNP} \beta_{SNP} + \epsilon, $$

where *X*_*SNP*_ is the design matrix for the genetic marker under study and *β*_*SNP*_ is the regression coefficient, also known as SNP effect. Every SNP genotype *X*_*SNP*_ was recoded using single numeric variables as (0, 1, 2), (0, 1, 1), (0, 0, 1) and (0, 1, 0) for testing potential additive, dominance, recessive, and overdominance effects, respectively. These models are also known as 1-degree-of-freedom models^14^. Note that these models assume that ${\varvec{\upepsilon}} \sim N(0,\;{\mathbf{V}}_{0} \sigma_{e}^{2} )$. The significance of the SNP effect under consideration was evaluated using the following test statistic:

$$ {\mathbf{z}} = \frac{{{\mathbf{X}}^{\prime}_{SNP} {\mathbf{V}}_{0}^{ - 1} \left( {{\mathbf{y}} - {\mathbf{X}}{\hat{\beta }}} \right)}}{{\sqrt {{\mathbf{X}}^{\prime}_{SNP} {\varvec{V}}_{0}^{ - 1} {\mathbf{X}}_{SNP} } }} $$

which approximates the Wald test, and hence, is asymptotically standard normal. These analyses were performed using the *R* package MixABEL^15^.

### Gene-set analysis

Following Han and Peñagaricano^16^, a three-step SNP-based gene-set analysis was implemented:

#### Assignment of SNPs to genes

The first step in the gene-set analysis consisted of the assignment of SNP markers to bovine genes. The Bioconductor *R* package biomaRt and the most recent bovine genome annotation (ARS-UCD-1.2) were used to map SNPs to genes. Specifically, SNPs were assigned to genes if they were located within the genomic sequence of an annotated gene or within 5 kb either upstream or downstream the gene. The distance of 5 kb was used to capture proximal regulatory regions and other functional sites that may lie outside (e.g., promoter region) but close to each gene. If a SNP was found to be located within or close to more than one gene, all these genes were included in subsequent analyses. Finally, a gene was considered as associated with Brown Swiss bull fertility if that gene contained at least one SNP with a significant additive effect (*P*-value ≤ 0.01).

#### Assignment of genes to functional gene-sets

Different functional databases, including Gene Ontology, KEGG pathways, Medical Subject Headings (MeSH) and InterPro, were used to define functional sets of genes. Genes assigned to the same functional term can be considered as members of a group of genes (gene-set) that share some properties, typically their involvement in the same biological process or molecular function.

#### Pathway-based association analysis

The significant association of a given functional term with bull fertility was assessed using the Fisher’s exact test, a test of proportions based on the cumulative hypergeometric distribution. This test was performed to investigate a potential overrepresentation of significant genes in each gene-set term. The *P*-value of observing *k* significant genes in the term was calculated by

$$ P{\text{-}} value = 1 - \mathop \sum \limits_{i = 0}^{k - 1} \frac{{\left( {\begin{array}{*{20}c} S \\ i \\ \end{array} } \right)\left( {\begin{array}{*{20}c} {N - S} \\ {m - i} \\ \end{array} } \right)}}{{\left( {\begin{array}{*{20}c} N \\ m \\ \end{array} } \right)}} $$

where *S* is the total number of significant genes associated with bull fertility, *N* is the total number of genes tested, and *m* is the total number of genes in the gene-set term. This overrepresentation analysis was performed using the *R* package EnrichKit, developed by Lihe Liu and Francisco Peñagaricano, available at https://github.com/liulihe954/EnrichKit.

### Functional gene-sets in common across different dairy breeds

Gene-set analyses were also performed using bull fertility records extracted from Holstein and Jersey breeds. For Holstein, we reanalyzed SCR records from 11,280 US Holstein bulls with 290,071 SNP markers^16^. For Jersey, we reanalyzed a dataset containing SCR records and 88,499 SNP markers from 1487 bulls^17^. The goal was to identify functional gene-sets, biological processes and molecular mechanisms that explain part of variation observed in dairy bull fertility in the three dairy breeds.

## Results and discussion

Improving reproductive performance remains a major goal in dairy cattle worldwide. Several studies have demonstrated that paternal factors play a major role on pregnancy establishment. Recently, our group developed a bull fertility evaluation in the Italian Brown Swiss cattle population using confirmed pregnancy records. We evaluated more than 1200 Brown Swiss bulls based on cow field data, including 400k breeding records from 130k lactating cows. Interestingly, we found a substantial variation in conception rate among Brown Swiss bulls, with more than 20% conception rate difference between high-fertility and low-fertility bulls^11^. As such, the present study was specially performed to identify genetic factors that explain part of the variation observed in sire conception rate among Italian Brown Swiss bulls.

### Whole genome scan: additive effects

**Figure 1:**
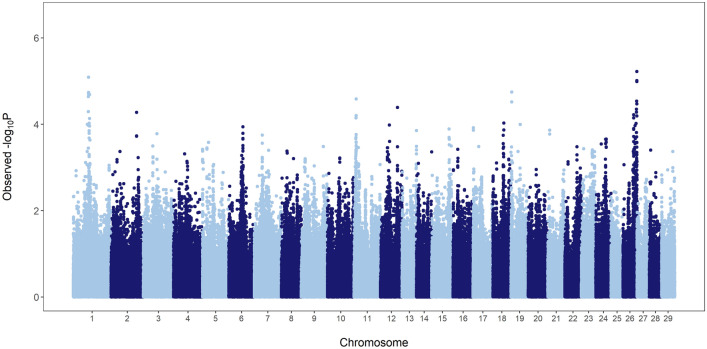
Manhattan plot showing the significance of additive effects on sire conception rate in Brown Swiss cattle.

The importance of additive effects on service sire fertility was evaluated on a genome-wide scale using a two-step mixed-model-based approach. Figure 1 shows the results of the genomic scan under the additive model. One genomic region on BTA1 showed significant effects on Brown Swiss bull fertility. The most significant marker, *rs43239680*, is located within an intron of gene *RABL3*. Interestingly, *RABL3* plays an important role in cell proliferation and cell motility^18^. It has been also shown that *RABL3* is vital for embryonic development, as the complete ablation of this gene in mice leads to embryonic lethality^19^. Overall, our results suggest that genetic variation in *RABL3* may explain part of the variation observed in male fertility in the Brown Swiss breed.

### Whole genome scan: non-additive effects

It is believed that non-additive effects are important for fitness-related traits such as fertility. Here, the potential role of non-additive effects on sire fertility in Brown Swiss cattle was evaluated using three different 1-degree-of-freedom tests corresponding to complete dominance, complete recessive, and pure overdominance effects. Alternatively, we could have used a genotypic model with two degree-of-freedom that fits simultaneously additive and non-additive effects. Note that if one is interested in identifying pure non-additive effects, namely complete dominance/recessive or pure overdominance, then the use of 1-degree-of-freedom tests is recommended because it maximizes the statistical power^20^. In addition, the 1-degree-of-freedom model facilitates the interpretation of the results because the *β*_*SNP*_ directly estimates the expected phenotypic difference between genotypic classes.

**Figure 2:**
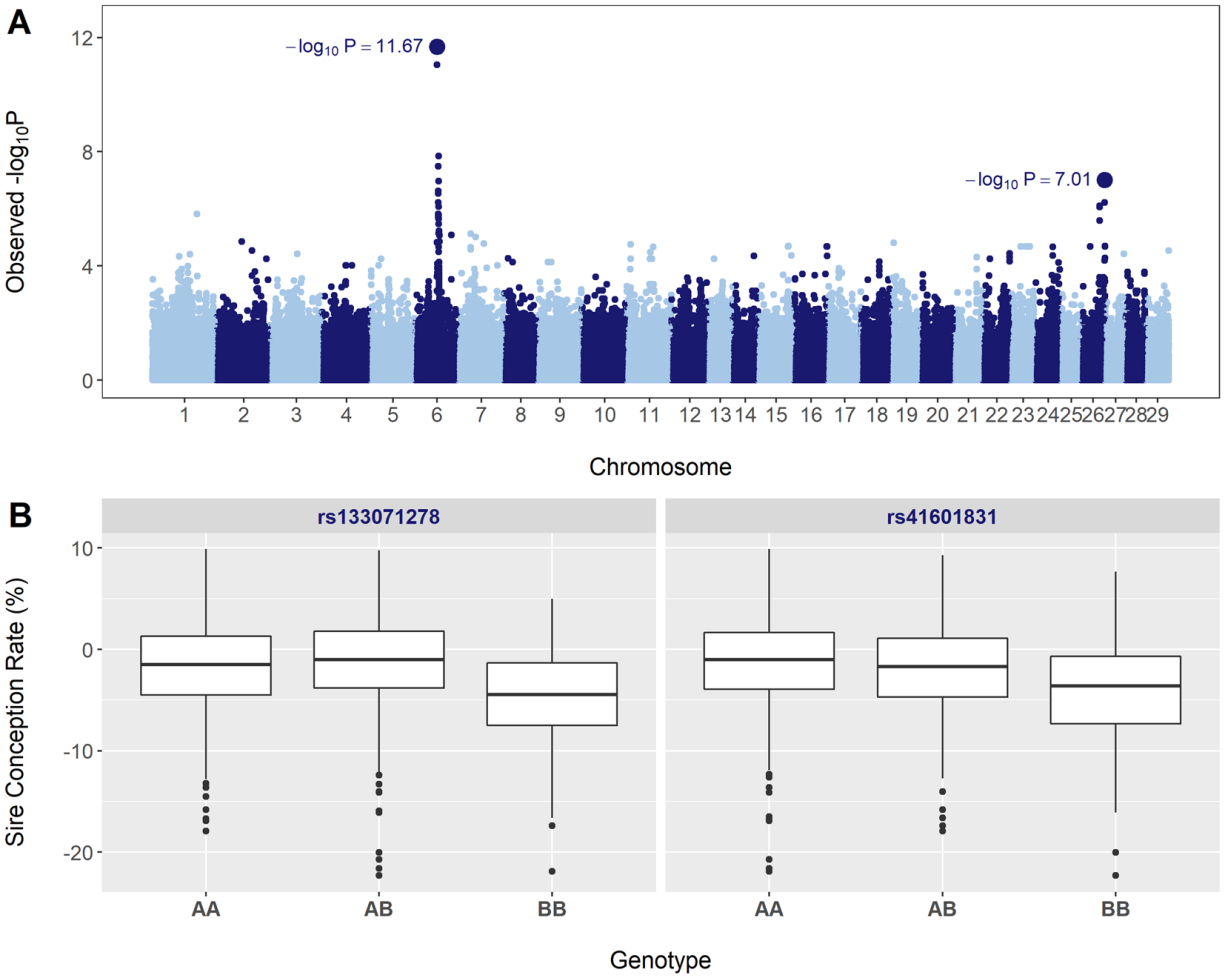
The relevance of non-additive effects on male fertility in Brown Swiss cattle. (**A**) Manhattan plot showing the significance of recessive effects. (**B**) Box plot showing the distribution of sire conception rate values for the two SNP loci with marked recessive effects.

We identified two genomic regions, located on BTA6 (57.6 Mb) and BTA26 (50.7 Mb), with significant recessive effects on sire conception rate (Fig. 2A). The distribution of sire conception rate values for the two SNP loci with marked recessive effects, *rs133071278* and *rs41601831*, is shown in Fig. 2B. Notably, these box plots show that the BB genotypes have much lower sire conception rate values than do genotypes AA and AB. Each of these loci explain differences in conception rates of around 3–4%. Unsurprisingly, the BB genotypes are in low frequency in the population, 8.4% and 10% for BTA6 and BTA26, respectively. No region showed complete dominance or pure overdominance effects.

The significant region detected on BTA6 harbors the gene *WDR19,* a very strong candidate gene for service sire fertility in Brown Swiss cattle. Indeed, previous studies using bull fertility data from Swiss, German, and Austrian Brown Swiss cattle populations identified a synonymous variant in *WDR19* significantly associated with various semen traits, including sperm motility and sperm abnormalities, and insemination success^21,22^. Gene *WDR19* is a constituent of the intraflagellar transport complex that is essential for the physiological function of motile cilia and flagella, including sperm motility^23^. Moreover, the significant region on BTA26 harbors the gene *ADGRA1* which encodes a protein that belongs to the adhesion family of G-protein-coupled receptors^24^. Of special interest, this family of receptors plays an important role in the fertilization process, inducing the acrosome reaction in bovine sperm^25^.

Recently, Hiltpold and collaborators reported that autosomal recessive loci contribute substantially to quantitative variation in bull fertility in Brown Swiss cattle^26^. Therefore, our study provides further evidence for the importance of non-additive effects in male fertility in cattle. Our findings confirm that genetic variation in *WDR19* is associated with reduced male fertility in Brown Swiss cattle. In addition, our results indicate that the region on BTA26 that harbors *ADGRA1* explains part of the variation observed in male reproductive performance among the Italian Brown Swiss bulls.

### Gene-set analysis

Genomic scans are powerful tools to detect genetic variants affecting quantitative traits. However, genomic scans typically detect only major variants, while most of the genetic variation remains hidden. Thus, complementary approaches are needed to fully reveal the genetic basis underlying a complex trait such as male fertility in cattle. Here, a gene-set analysis was performed to identify biological processes and molecular mechanisms responsible for the variation in bull fertility in the Italian Brown Swiss population.

**Figure 3:**
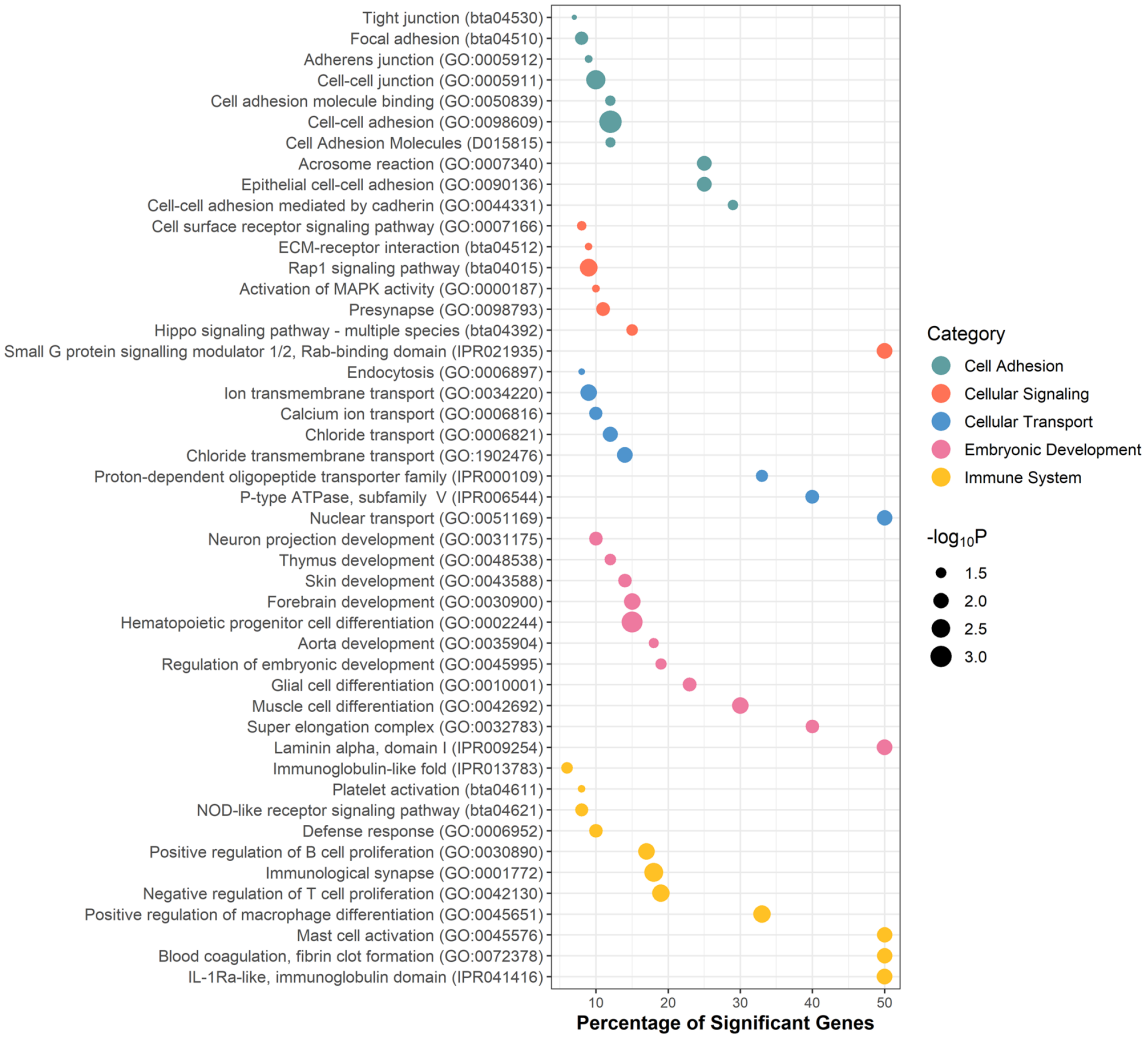
Functional gene-sets significantly enriched with genes associated with sire conception rate in Brown Swiss cattle. The y-axis displays the names of the gene-sets, the size of the dots represents the significance of the enrichment (− log_10_ *P*-value, Fisher’s exact test) and x-axis represents the percentage of significant genes in each gene-set.

Figure 3 shows the most relevant biological terms and pathways associated with service sire fertility. A total of 231,764 of the 454,556 examined SNP markers were located within or near 22,467 annotated genes in the bovine reference genome ARS-UCD-1.2. A subset of 833 genes were defined as significant associated with bull fertility given that contained at least one significant SNP. Our gene-set analysis interrogated different gene-set databases, including GO, KEGG, MeSH and InterPro. Across these databases, genome-wide association signals for service sire fertility were highly enriched in at least five groups of gene-sets, namely cell adhesion, cellular signaling, cellular transport, embryonic development, and immune system.

Our enrichment analysis revealed several significant functional terms related to cell adhesion. Note that most of the events that occur before and during the fertilization process, including gametogenesis, gamete transport, and sperm-oocyte interaction, involve cell adhesion events. Therefore, the impaired function of genes involved in cell adhesion might result in early pregnancy failures^27,28^. Cellular signaling and cellular transport pathways, such as activation of MAPK activity (GO:0000187) and calcium ion transport (GO:0006816), are also involved in many processes related to spermatogenesis and early embryo development^29,30^. Gene-sets involved in embryonic development, including muscle cell differentiation (GO:0042692) and super elongation complex (GO:0032783), were also associated with variation in bull fertility. These findings provide further evidence that paternal factors contribute to early embryo development in cattle^31^. Finally, gene-sets directly related to the immune system showed a significant enrichment of genes associated with bull fertility. The immune system impacts pregnancy establishment in different ways, from the spermatogenesis in the male through the fertilization in the female reproductive tract^32^.

Overall, a successful pregnancy establishment requires a very well-orchestrated cascade of events. Our findings show that genetic variation underlying different processes, including cell adhesion, cell motility, and the immune response, explain part of differences observed in male fertility in Brown Swiss cattle.

### Functional terms in common across different dairy breeds

**Figure 4:**
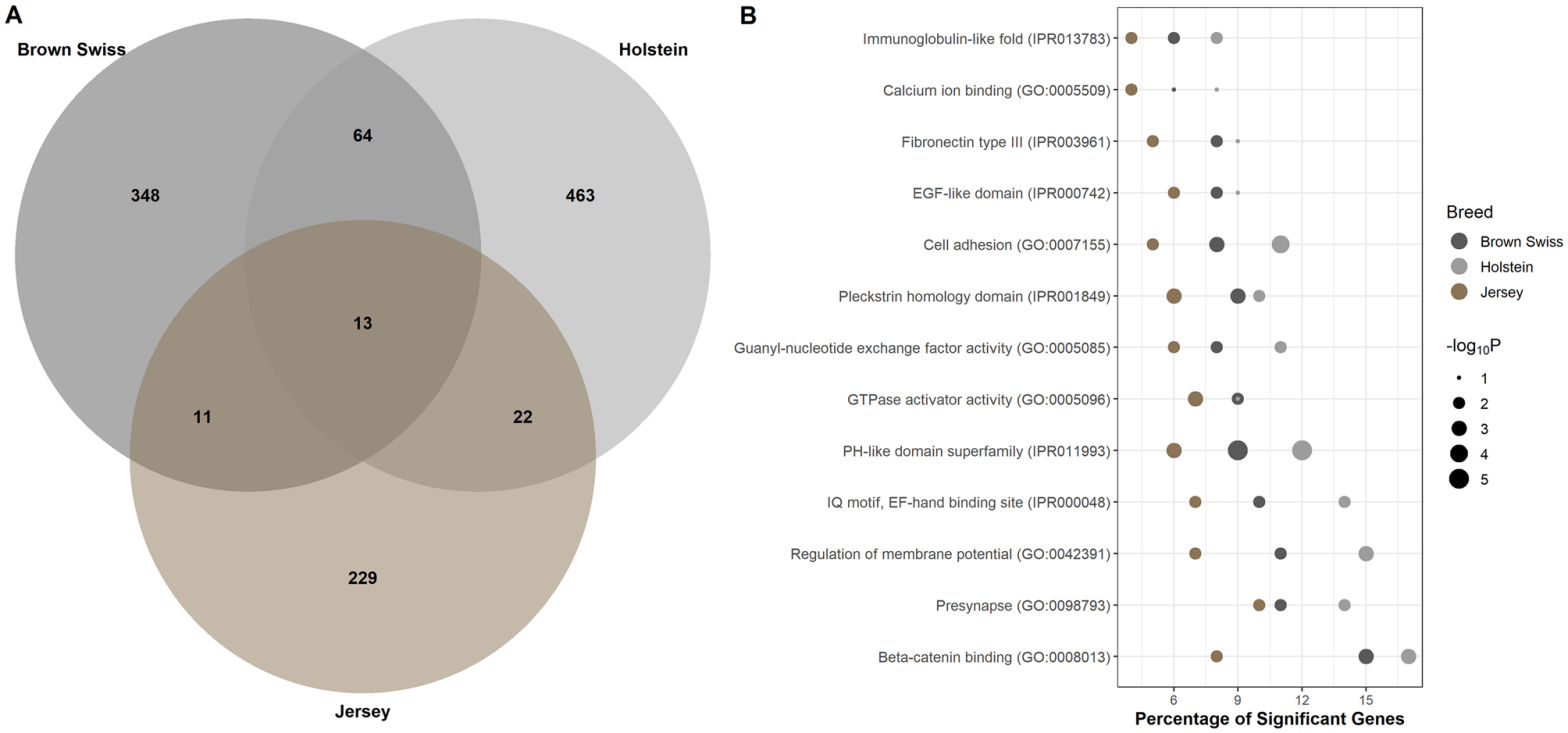
Functional gene-sets associated with male fertility across different dairy breeds. (**A**) Venn diagram showing the number of gene-sets significantly associated with sire conception rate in Brown Swiss, Holstein, and Jersey cattle. (**B**) Significant gene-sets identified in the three dairy breeds under study. The y-axis displays the names of the gene-sets, the size of the dots represents the significance of the enrichment (− log_10_ *P*-value, Fisher’s exact test) and x-axis represents the percentage of significant genes in each gene-set.

None of the genomic regions or individual genes identified in this study were previously reported as significantly associated with male fertility neither in Holstein nor in Jersey. This may be due to multiple causes, namely the major genetic variants affecting male fertility in Brown Swiss are not segregating in Holstein or Jersey, or these major genetic variants are segregating in these two dairy breeds but are not in high linkage disequilibrium with the markers in the SNP chips or simply false-positive/false-negative results. Notably, the gene-set analysis performed across the three dairy breeds identified a set of functional terms that are associated with male fertility in all the breeds (Fig. 4). These gene-sets are involved in cell migration, such as Fibronectin type III (IPR003961), cell–cell interaction, such as cell adhesion (GO:0007155) and beta-catenin binding (GO:0008013), cellular signaling, such as calcium ion binding (GO:0005509) and PH-like domain superfamily (IPR011993), GTPase activity, such as Guanyl-nucleotide exchange factor activity (GO:0005085) and GTPase activator activity (GO:0005096), and the immune response, such as Immunoglobulin-like fold (IPR013783). Supplementary Table 2 reports the full list of significant biological terms for each breed, including term name and ID, P‐value, total number of genes, number of significant genes and database. Remarkably, these results demonstrate that biological processes and molecular pathways, rather than single genes, are the primary targets of selection.

## Conclusions

We performed an integrative genomic analysis to better understand the genetic basis of male reproductive performance in Brown Swiss cattle. We identified genomic regions in BTA1, BTA6, and BTA26, associated with sire conception rate. These regions harbor genes, such as *RABL3*, *WDR19* and *ADGRA1*, with known roles in sperm biology and fertilization. We also performed a gene-set analysis to gain additional insights into the genetics of male fertility in cattle. Our analysis using Brows Swiss data identified functional terms involved in cell adhesion, cellular signaling, cellular transport, embryonic development, and immune system. Remarkably, a gene-set analysis also including Holstein and Jersey data, revealed significant processes that are common to the three dairy breeds, including cell migration, cell–cell interaction, GTPase activity, and the immune function. Overall, this study sheds light on the genetic variants and mechanisms that explain part of the variation observed in dairy bull fertility. These findings may contribute to the development of novel strategies for improving service sire fertility, including the use of biologically informed genomic prediction models.

## Supplementary Information

Supplementary Table 1.

Supplementary Table 2.

## Data Availability

The phenotypic and genotypic data analyzed in this study were obtained from Italian Brown Breeders Association. These datasets were used under agreement, and hence, are not publicly available. However, data are available upon request to Francisco Peñagaricano and with permission of the Italian Brown Breeders Association.
